# Volume‐localized measurement of oxygen extraction fraction in the brain using MRI

**DOI:** 10.1002/mrm.27823

**Published:** 2019-05-27

**Authors:** Caitlin O'Brien, Thomas W. Okell, Mark Chiew, Peter Jezzard

**Affiliations:** ^1^ Wellcome Centre for Integrative Neuroimaging, FMRIB Division, Nuffield Department of Clinical Neurosciences University of Oxford Oxford United Kingdom

**Keywords:** hematocrit, non‐invasive measurement, oxygen extraction fraction, oxygen saturation, TRUST

## Abstract

**Purpose:**

T_2_‐relaxation‐under‐spin‐tagging (TRUST) is an MR technique for the non‐invasive assessment of whole‐brain cerebral oxygen extraction fraction (OEF), through measurement of the venous blood T_2_ relaxation time in the sagittal sinus. A key limitation of TRUST, however, is the lack of spatial specificity of the measurement. We sought to develop a modified TRUST sequence, selective localized TRUST (SL‐TRUST), having sensitivity to venous blood T_2_ within a targeted brain region, and therefore achieving spatially localized measurements of cerebral tissue OEF, while still retaining acquisition in the sagittal sinus.

**Methods:**

A method for selective localization of TRUST sequence was developed, and the reproducibility of the technique was evaluated in healthy participants. Regional measurements were achieved for a single hemisphere and for a 3D‐localized 70 × 70 × 80 mm^3^ tissue region using SL‐TRUST and compared to a global TRUST measure. An additional measure of venous blood T_1_ in the sagittal sinus was used to estimate subject‐specific hematocrit. Six subjects were scanned over 4 sessions, including intra‐session repeat measurements.

**Results:**

The average T_2_ in the sagittal sinus was found to be 60.8 ± 8.9, 62.7 ± 7.9, 64.6 ± 8.4, and 66.3 ± 10.3 ms (mean ± SD) for conventional TRUST, global SL‐TRUST, hemispheric SL‐TRUST, and 3D‐localized SL‐TRUST, respectively. Intra‐, inter‐session, and inter‐subject coefficients of variation for OEF using SL‐TRUST were found to be comparable and in some cases superior to those obtained using TRUST.

**Conclusion:**

OEF comparison of 2 contralateral regions was achievable in under 5 min suggesting SL‐TRUST offers potential for quantifying regional OEF differences in both healthy and clinical populations.

## 1 INTRODUCTION

The oxygen extraction fraction (OEF) is the relative difference in oxygen concentration between arterial (Y_a_) and venous (Y_v_) blood, $\text{OEF}\left(\%\right)=\frac{Y_{a}-Y_{v}}{Y_{a}}\;\times 100$ (also expressible as a fraction $\text{OEF}\;=\frac{Y_{a}-Y_{v}}{Y_{a}}$), and represents the proportion of oxygen extracted by tissue as blood passes through the capillaries. Changes in OEF reflect underlying changes in oxygen metabolism of the tissue and can be an indicator of cell stress or death in normal aging or cerebrovascular conditions such as Alzheimer disease, Parkinson disease, and ischemic stroke.^1^, ^2^, ^3^, ^4^ Reliable quantification and mapping of cerebral oxygenation, along with other physiological parameters, such as cerebral blood flow (CBF), would allow better understanding of both normal and abnormal brain physiology. However, a lack of robustness and accessibility has hindered clinical application of currently available methods. PET is considered the current gold standard for cerebral OEF mapping, although the requirement of short‐lived O‐15 radiotracers leaves it undesirable for research and limits accessibility for clinical application.^5^, ^6^, ^7^ Some focus has therefore shifted toward MRI to provide a solution to quantifying cerebral OEF non‐invasively.

MRI‐based methods include calibrated fMRI,^8^ susceptibility‐based oximetry,^9^ use of velocity selective gradient filters to isolate venous blood signal,^10^ and quantitative BOLD imaging.^11^ Often, however, the methods lack spatial specificity, struggle to isolate venous signal from surrounding static tissue or lack accuracy, suffer from low SNR, or are highly sensitive to B_0_ inhomogeneities or model parameters.

In 2008, Lu and Ge^12^ devised an MR method, T_2_‐relaxation‐under‐spin‐tagging (TRUST), which uses a spin labeling preparation to isolate and measure venous blood T_2_ in the sagittal sinus. Because of a known relationship between blood T_2_ and blood oxygen saturation levels, this then allows calculation of tissue OEF. The sensitivity of TRUST to changes in OEF has been demonstrated using hypercapnia, hyperoxia, hypoxia, and caffeine challenges.^12^, ^13^, ^14^, ^15^ The robustness and applicability of the method has been demonstrated further in a number of single and multi‐site studies and in a variety of cerebrovascular conditions.^16^, ^17^, ^18^ Overall, the TRUST method has been highly influential, however, a key limitation of the method is its lack of spatial specificity.

Here, we adapt the original TRUST sequence such that spatially specific measures of venous blood T_2_ can be achieved and therefore quantify OEF across different regions of the brain. The method, denoted selective localized TRUST (SL‐TRUST), performs the T_2_‐encoding while the venous blood still resides close to the brain tissue it has drained from, making it sensitive to local OEF changes, and uses water suppression enhanced through T_1_ effects (WET)^19^ spatially selective saturation pulses to isolate venous blood signal from localized regions of the brain. This signal is later decoded in the superior sagittal sinus,^20^, ^21^ and background suppression methods are used to minimize tissue subtraction errors.^22^ The robustness and reproducibility of SL‐TRUST is assessed in whole brain (and compared to whole brain conventional TRUST measurements), as well as in a single hemisphere, and in 70 × 70 × 80 mm^3^ tissue regions in healthy participants. After demonstrating the basic method the inter‐ and intra‐scan coefficient of variation (CoV) is calculated for each measurement, along with inter‐subject variability. The calculation of tissue OEF is improved further with an additional venous blood T_1_ measurement, also in the sagittal sinus, to estimate subject‐specific hematocrit (HCT) levels, rather than assuming uniform HCT across all subjects.

## 2 METHODS

### 2.1 Theory

A higher fraction of deoxyhemoglobin in blood leads to enhanced signal attenuation by T_2_ relaxation. The relationship between the transverse relaxation time T_2_ and blood oxygenation has been derived from a model that uses a compartment‐weighted sum of relaxation rates from plasma and hemoglobin contributions inside the erythrocytes of blood,^23^ which simplifies to:

(1) $$\frac{1}{T_{2}}=A+B\;\cdot \;\left(1-Y_{v}\right)+\;C\;\cdot \;{\left(1-Y_{v}\right)}^{2},$$

where Y_v_ is the oxygen saturation fraction in venous blood. Assuming the arterial blood to be fully oxygenated (Y_a_ ≈ 1), and relatively homogeneous across healthy individuals, Equation 1 simplifies further to:

(2) $$\frac{1}{T_{2}}=A+B\;\cdot \;\text{OEF}+C\;\cdot \;{\text{OEF}}^{2},$$

where A, B, and C are coefficients that depend on the blood HCT (the volume percentage of red blood cells [ HCT]):

$$A=\;a_{1}+\;a_{2}\;\cdot \;\text{HCT}+\;a_{3}\;\cdot \;{\text{HCT}}^{2}$$

$$B=\;b_{1}\;\cdot \;\text{HCT}+\;b_{2}\;\cdot \;\;{\text{HCT}}^{2}$$

(3) $$C=\;c_{1}\;\cdot \;\text{HCT}\;\;\cdot \;\left(1-\text{HCT}\right),$$

in which $a_{1..n},\;b_{1..n},\;c_{1}$ are also dependent on the MR sequence parameters and have been derived using oxygenation‐ and temperature‐controlled bovine blood experiments.^24^, ^25^ The blood HCT can be determined from a blood sample. However, where this is not available, the HCT is often assumed to be uniform across subjects, HCT = 0.4 and 0.43 for female and male participants, respectively.^16^, ^26^, ^27^

Alternatively, there exists a linear dependency between the longitudinal relaxation rate, R_1_, and blood HCT, which allows HCT to be estimated non‐invasively. A number of different calibration equations exist for blood T_1_ and HCT. Most recently, Shimada et al^28^ presented an in vivo calibration for venous blood T_1_ and HCT, measured at 3T in the internal jugular vein, yielding:

(4) $$\frac{1}{T_{1}}=\left(0.70\;\pm \;0.11\right)\;\cdot \;\text{HCT}+\left(0.27\;\pm \;0.05\right)\;s^{-1}.$$

Therefore, by using a venous blood T_1_ measurement, one may calculate venous blood HCT and, therefore, when combined with a measure of venous blood T_2_, estimate tissue OEF.

### 2.2 TRUST

The original TRUST method applies a spin‐labeling inversion to the venous side of the vasculature by inverting the magnetization in a tissue slab (rather than by inverting blood in the arterial side as is done in conventional arterial spin labeling [ASL]) and, after an inversion delay TI, a readout slice placed through the superior sagittal sinus is used to measure the signal of the outflowing venous blood spins. Pairwise subtraction of labeled and unlabeled images reveals the signal present in the sagittal sinus arising from tagged venous blood spins.

Before image acquisition, a series of non‐slice‐selective global T_2_‐preparation pulses modulate the venous blood signal by applying different T_2_‐weightings across different measurements according to a series of effective echo times (eTE). Within the T_2_‐preparation module, a train of 180° pulses helps to mitigate unwanted pseudo‐T_2_ effects arising from diffusion through microscopic field gradients surrounding the deoxyhemoglobin. The time between consecutive pulses is referred to as the inter‐echo time (T_CPMG_). The coefficients *a*_1_, *b*_1_, etc. in Equation 3 have been derived for a range of inter‐echo durations, T_CPMG_ = 2–20 ms.^24^, ^25^ Throughout this study, an inter‐echo duration of T_CPMG_ = 10 ms is used, therefore the coefficients in Equation 3 are as follows: a_1_ = −13.5, a_2_ = 80.2, a_3_ = −75.9, b_1_ = −0.5, b_2_ = 3.4, and c_1_ = 247.4 (s^−1^).^25^

Following pairwise subtraction, and for a specific inversion delay, TI, the resulting pure venous blood signal measured in the sagittal sinus, (ΔS), is given by:

(5) $$\Delta S=S_{0}e^{eTE\left(\frac{1}{T_{1v}}-\frac{1}{T_{2v}}\right)}.$$

Where S_0_ is the pure venous blood signal when no T_2_ weighting is applied, and T_1v_ is fixed at a representative value of 1612 ms, derived from R_1v_ = 0.62 s^−1^ (for a complete derivation, see reference).^29^

### 2.3 Selective localized TRUST

**Figure 1:**
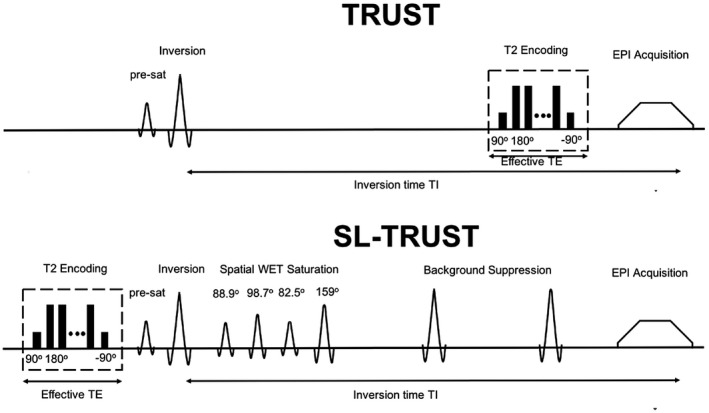
Comparison of T_2_‐relaxation‐under‐spin‐tagging (TRUST) and selective localized TRUST (SL‐TRUST) MR pulse sequence diagrams. Interleaved acquisitions of label and control scans, achieved using an on–off resonance inversion pulse. Each image type is acquired with 6 different effective echo times (eTE = 0, 40, 80, 120, 160, 200 ms), each performed twice, and averaged to mitigate pulsatile effects. In TRUST, the T_2_‐encoding is performed directly before the read out whereas, in the case of SL‐TRUST this T_2_‐encoding occurs before the inversion. An inversion delay TI = 1200 ms allows labeled blood to flow into the imaging slice. In SL‐TRUST, spatial saturation and background suppression pulses are inserted between the inversion and the EPI acquisition

The initial modification made in this study to the original TRUST method to provide spatial selectivity in the OEF measurement is the relocation of the T_2_‐preparation module (Figure 1). In conventional TRUST, the T_2_‐weighting is applied immediately before the readout, and therefore the T_2_ measured in the sagittal sinus is dependent on the oxygenation of blood within this vein, representing the average OEF across all brain regions that drain into it. In contrast, in SL‐TRUST the T_2_‐weighting is performed at the start of the sequence, before the inversion pulse. Therefore, the local venous T_2_, and therefore OEF, are encoded in the longitudinal magnetization of the venous blood. When spatial saturation pulses are inserted following the inversion pulse, only spins from venous blood within a defined brain region contribute to the measured signal in the sagittal sinus at the time of the readout, which is modulated by the T_2_‐weighting applied previously. Therefore, the measured signal relates to the venous T_2_ in the unsaturated (retained) brain region, rather than the T_2_ of venous blood within the sagittal sinus itself. In this manner, the regional localization of signal is achieved while maintaining a high‐partial‐volume readout in the sagittal sinus.

### 2.4 Spatial localization

To achieve spatial specificity, spatial saturation pulses are inserted immediately after the labeling inversion pulse. Two slice‐selective WET saturation schemes are used to saturate all spins in 2 orthogonal specified regions. Based on n‐pulse variable‐angle methods, WET was developed as a water suppression method in MR spectroscopy.^19^ The scheme used here is optimized as a gradient‐selective saturation method and uses 4 varying flip angles (88.9°, 98.7°, 82.5°, and 159°) to achieve zero longitudinal magnetization for a range of T_1_ values and robustness against B_1_ field inhomogeneity.^30^ Additionally, each RF‐pulse in the WET pulse train is cosine modulated so as to separate out a single saturation band into 2 parallel saturation regions that straddle the region that is to be retained (in practice, the 2 required sub‐pulses were designed separately and summed). To avoid voltage clipping on the scanner because of the additive nature of the 2 component modulated pulses, a small time shift was inserted between them, extending the total pulse duration by 25%. The region to be retained was graphically prescribed using a maximum of 2 WET saturation schemes (therefore giving 4 saturation bands). The selective nature of the tagging inversion pulse provides the spatial extent in the third dimension. In this manner, a volume of interest can be selected (retained), analogous to the rectilinear volume regions assessed in localized spectroscopy.

### 2.5 Background suppression

In the original TRUST method, the T_2_‐encoding module immediately before the readout acts to help suppress the static tissue signal at longer eTE values, therefore even if the pairwise subtraction of tag‐control pairs is imperfect, the unwanted contribution from the static tissue signal reduces for longer eTEs. In SL‐TRUST, however, any unwanted contribution from the static tissue signal will not reduce for longer eTEs, because of moving the T_2_‐encoding module temporally further away from the readout, which in turn risks affecting the likelihood of subtraction errors (and hence effective SNR) in the readout signal of interest at long eTE values. This can manifest as signal fluctuations in the T_2_ decay curves, which is aggravated further when the venous blood signal is generated from a smaller region and therefore the total signal available is reduced. To circumvent this, a multiple inversion recovery (MIR) background suppression method, ASSIST,^22^ was used to null the static tissue signal at the time of the read out. Two nonselective inversion pulses are played out at times τ_1_ and τ_2_ relative to the labeling pulse (and also requires a selective nulling of the signal in the plane of the readout pulse at the time of the labeling pulse). τ_1_ and τ_2_ are calculated such that components with relaxation rates, R_1opt_ and 0.5 × R_1opt_, are nulled at the time of the readout^31^:

(6) $$\tau_{1,2}\left(\text{TI}\right)\;=\;\text{TI}+\frac{2}{R1_{\text{opt}}}\log\left(\left(0.5\;\pm \;0.25\right)+\left(0.5\;\mp \;0.25\right)e^{-0.5\;\cdot \;\text{TI}\;\cdot \;R1_{\text{opt}}}\right).$$

### 2.6 Sequence timings

An optimal inversion delay, TI, has been reported for TRUST as being 1200 ms for a 20 mm gap between the bottom of the labeling slab and the read out slice.^12^ However, in the presence of saturation bands in SL‐TRUST, spins closest to the imaging slice may have been saturated, therefore the initial signal peak from inflowing spins may be delayed relative to the TRUST method. Therefore, using a series of incrementing inversion delays (TI = 100–1200 ms), we investigated whether TI = 1200 ms was still an appropriate inversion delay to use for the SL‐TRUST method.

Similarly, the scan TR used in the original TRUST sequence was TR = 8 s.^12^ In subsequent publications, this has been reduced to TR = 3 s^29^ with the use of a post‐saturation pulse. A shorter TR is desirable to reduce the overall scan time but risks introducing a systematic bias and overestimating T_2_ because of residual effects on the magnetization remaining from the global T_2_‐preparation pulses of previous TRs.^12^, ^29^ We hypothesized that because of the T_2_‐preparation pulses occurring much earlier in the SL‐TRUST sequence, there are reduced residual magnetization effects in subsequent TRs and therefore a shorter TR can be achieved without such adverse effects. To assess this, we investigated and compared T_2_ values estimated using 3 TR values, 8 s, 6 s, and 4 s.

**Figure 2:**
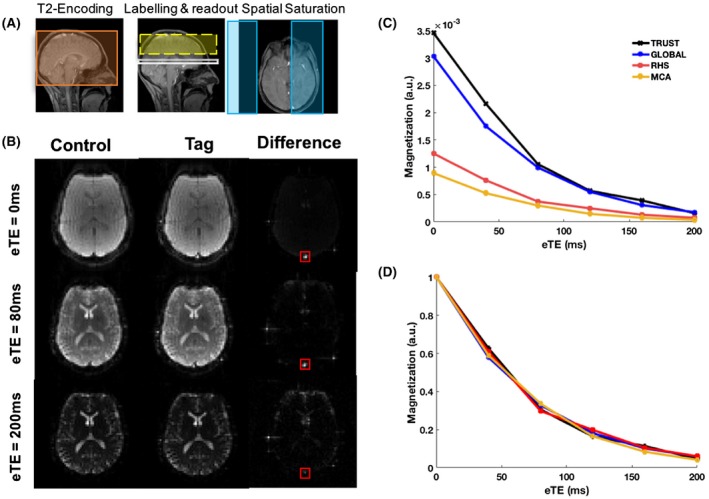
A, Illustration of the placement of the global T_2_‐encoding (orange), the labeling inversion slab (yellow), the read out slice (white), and hemispherical spatial saturation (blue). Note that for more regional selection a second pair of spatial saturation pulses are added orthogonally to those shown in blue. B, Example of tag, control, and resulting difference images, with the ROI around the sagittal sinus (red), for varying effective echo times. C, Example difference signals as a function of eTE in a single subject for TRUST, and SL‐TRUST where the signal arises from the whole brain (global), just 1 hemisphere (RHS), and just the right middle cerebral artery territory (MCA), demonstrating the signal loss because of WET spatial saturation. D, Normalized curves

The full sequence diagrams comparing TRUST and SL‐TRUST can be seen in Figure 1, with the geometric positioning of key sequence components, example tag, control, and resulting difference images, and the difference signal as a function of effective echo time eTE shown in Figure 2.

### 2.7 T_1_ measurement for HCT estimation

The relationship between the longitudinal relaxation time T_1_ and the HCT of blood, given by Equation 4, was used to estimate subject‐specific HCT non‐invasively. A multi‐TI inversion recovery sequence was used to measure venous blood T_1_ in the superior sagittal sinus. A tag‐control global inversion, similar to the method used in SL‐TRUST, enables pairwise subtraction, leaving only the inverted venous blood spins contributing difference signal, and is repeated for multiple inversion delays. To null the static tissue contributions further, background suppression, in the form of 2 slice selective 180° pulses through the read‐out slice, was used, provided the inversion delay was sufficiently long to accommodate the 2 pulses and τ_2_ was sufficiently short (far enough away from the readout) to ensure they did not disrupt the incoming venous spins.^22^

### 2.8 MRI experiments

Reproducibility data were acquired from 6 healthy volunteers (mean age 29.2 y, SD ±8 y, 3 males) under a technical development protocol approved by the local ethics committee. All experiments were performed on a 3T, Siemens Verio scanner (Siemens Healthineers, Erlangen, Germany), with a 32‐channel head receive coil and body transmit. Each participant underwent 4 separate scan sessions over the course of 2 weeks to assess the method reproducibility. The scan protocol for each session included a whole‐brain (global) conventional TRUST measurement a global SL‐TRUST measurement (i.e., no spatial saturation but intended to act as a direct comparison with the conventional TRUST measurement), a hemispherical SL‐TRUST measurement (i.e., a single pair of parallel spatial saturation pulses intended to retain signal from a brain hemisphere), and a SL‐TRUST measurement prescribed over a 70 × 70 × 80 mm^3^ tissue region located in the middle cerebral artery territory (i.e., 2 pairs of orthogonally prescribed saturation pulses intended to retain signal from a cuboid region, with the extent of the selective inversion pulse serving to provide the 80 mm third dimension). The order of scans in the protocol was randomized to avoid time‐dependent bias because of participant fatigue. In total, the protocol took ~9 min for all scans in each session. During the initial scan session, the protocol was repeated an additional 2 times, once without repositioning of the subject, and once after removing and repositioning the subject within the scanner. In each of the 3 follow‐up scan sessions, the protocol was repeated 1 additional time, without repositioning of the subject. During 1 of the 4 scan sessions, an additional venous blood T_1_ measurement was acquired using the multi‐inversion recovery sequence described above. Three T_1_ measurements were acquired during that scan session, an initial T_1_ measurement (later used to estimate subject‐specific HCT) and 2 follow up measurements, with and without repositioning of the subject, to calculate the inter‐ and intra‐scan coefficients of variability in T_1_. In all cases, the readout slice was angled to be parallel to the anterior commissure–posterior commissure line.

Experiments to optimize sequence timing parameters TI and TR were conducted separately in 2 different healthy participant groups, with n = 5 (mean age = 36 y, 2 males) and n = 4 (mean age = 39 y, 3 males) respectively.^20^

For TRUST and SL‐TRUST, an MLEV‐4 scheme^32^ with an inter‐echo spacing of T_CPMG_ = 10 ms was used for T_2_ encoding, giving an effective TE step size of 40 ms and therefore eTE = 0, 40, 80, 120, 160, and 200 ms.

Based on the results of the scan repeat time investigation (see below), TR = 8 s and 4 s was used for TRUST and SL‐TRUST, respectively. Other imaging parameters included: EPI TE = 23 ms, FOV = 240 mm, matrix = 64 × 64, slice thickness = 5 mm, gap between labeling slab and imaging slab = 20 mm, and 2 averages per measurement. Approximate scan time for a single T_2_ measurement is 2 min. For the multi‐TI measurement of T_1_, an initial TI = 100 ms was used, with ΔTI = 550 ms, TR = 10 s, slice thickness = 10 mm, 9 increments, and 2 averages, total scan time per T_1_ measurement was therefore ~4 min. Together, these give a total scan time of 45 min during the initial scan session and 20 min in each of the 3 follow‐up scan sessions.

### 2.9 Data analysis

Raw k‐space data were captured and processed using in‐house MATLAB (The MathWorks, Natick, MA) scripts, and the complex nature of the data maintained throughout analysis steps to account for polarity effects from the background suppression pulses. Because of the localized origin of the signal in the sagittal sinus vein and its proximity to the edge of the brain, an adaptive combine coil‐combination method was used to maximize the SNR^33^ following EPI phase correction and Fourier transformation. The images were inspected for motion, first visually, and then using the MCFLIRT FSL tool.^34^ MCFLIRT motion correction parameters showed a correction of ~0.3 mm that was not considered significant, however, interpolation and smoothing algorithms contained within MCFLIRT resulted in blurring of tissue signal into the region of interest (ROI) containing the blood vessel. Motion correction is challenging for 2D images with multiple saturation bands and such small identifiable motion (personal correspondence, FSL developers) and, therefore, with the added risk of inducing partial volume effects the cost of motion correction outweighed the benefits and was not performed.

Pairwise subtraction between the control and labeled images highlighted the superior sagittal sinus, around which a ROI was manually drawn. The 4 highest intensity pixels in the ROI of the highest contrast image, eTE = 0 ms for TRUST and SL‐TRUST, and TI = 100 ms for inversion recovery, were selected to create a mask that was applied to all further eTE or TI time points, respectively.^12^ Spatial averaging of the voxels yielded the venous blood signal at each time point, which was subsequently fitted to a mono‐exponential function to obtain T_2_ or T_1_. The T_2_ and T_1_ values were then converted to OEF and HCT, as appropriate, via Equations 2 and 4, respectively. The residuals of the fit were used to estimate the SD of the noise at each effective echo time. This information was used to perform Monte‐Carlo simulations (using 500 randomly generated curves) to estimate the uncertainty on the fit parameters.

### 2.10 Reproducibility

The following metrics were calculated for T_2_ and T_1_ to assess reproducibility.

First, intra‐session CoV (that reflects the measurement noise) was calculated as

(7) $$CoV_{intra-session}=\frac{1}{I\;\cdot \;J}\sum_{i}\sum_{j}\frac{\left|M_{ij1}-M_{ij2}\right|}{\sqrt{2}\;\cdot \;mean\left(M_{ij1},\;M_{ij2}\right)},$$

where *M_ijk_* represents measurement #*k* (*k* = 1, 2) of Subject #*i* (*i* = 1, 2, 3 … *I*) in Session #*j* (*j* = 1, 2, 3 … *J*).

Second, inter‐session CoV was calculated as

(8) $$CoV_{inter-session}=\frac{1}{I\;\cdot \;K}\sum_{i}\sum_{k}\frac{SD_{j}(M_{ijk)}}{mean_{j}\left(M_{\mathrm{ijk}}\right)},$$

where mean_j_ and SD_j_ are mean and SD across sessions, respectively.

Third, the inter‐subject CoV was calculated as

(9) $$CoV_{inter-subject}=\frac{1}{J\;\cdot \;K}\sum_{j}\sum_{k}\frac{SD_{i}\left(M_{\mathrm{ijk}}\right)}{mean_{i}\left(M_{\mathrm{ijk}}\right)}.$$

Subject repositioning and day‐to‐day physiology differences are contained within the inter‐session CoV. These contributions can be estimated using $\sqrt{{\text{CoV}}_{\text{inter}-\text{session}}^{2}-{\text{CoV}}_{\text{intra}-\text{session}}^{2}}$. Similarly, inter‐subject CoV contains physiological differences between subjects, which can be considerable. These can be estimated using $\sqrt{{\text{CoV}}_{\text{inter}-\text{subject}}^{2}-{\text{CoV}}_{\text{inter}-\text{session}}^{2}}$.^35^

## 3 RESULTS

Figure 2 shows example SL‐TRUST data from a single subject in comparison to conventional TRUST. The normalized T_2_‐decay curves (Figure 2D) demonstrate the comparable data quality and similarity of the observed T_2_ between TRUST and all the regional SL‐TRUST measurements. In addition, the signal differences observed in the unnormalized data (Figure 2C) indicate that the spatial saturation has been successful and signal localization is achieved. On average, a signal decrease of 64.5 ± 15.4% and 78.8 ± 11.5% was seen for hemispherical and MCA regions, respectively, in comparison to global SL‐TRUST. In the hemispherical case, this signal reduction is a little greater than the 50% that might be expected, however, this is likely because of the placement of the saturation bands to cover the sagittal sinus, therefore removing all contributions from blood initially within this large vein as well as saturating a small portion of the contralateral hemisphere. The effectiveness of the background suppression pulses was also investigated and was found to achieve an average 96.1 ± 0.6% decrease in the background static tissue signal (data not shown).^21^

**Figure 3:**
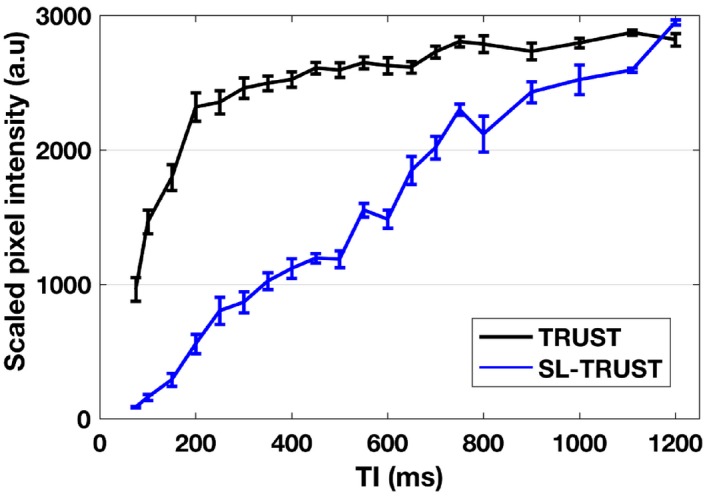
Tagged blood bolus arrival for a range of inversion delays (TI = 75–1200 ms) for TRUST and SL‐TRUST with spatial saturation through the sagittal sinus, averaged across 5 subjects. Saturating those spins already present in the superior sagittal sinus delays the arrival of the bolus in comparison to TRUST, however, both sequences achieve similar signal intensities at TI = 1200 ms

**Figure 4:**
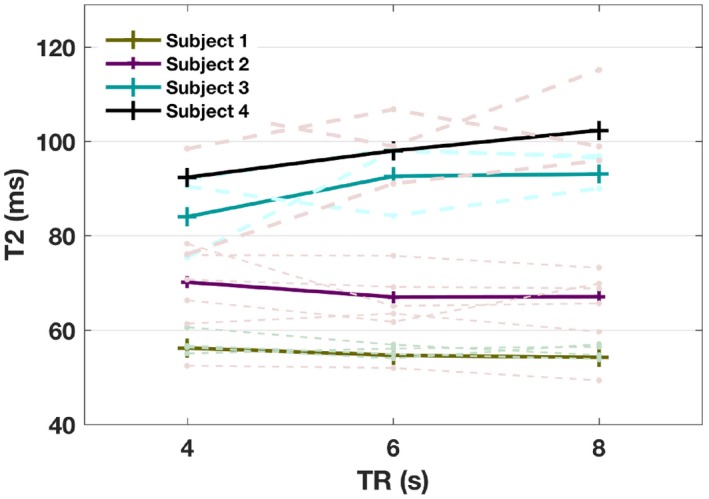
Repetition time dependence. T_2_ values in the sagittal sinus for global SL‐TRUST for a range of repetition times TR = 4, 6, 8 s at TI = 1200 ms. The average T_2_ values are shown with solid lines, with the underlying individual measurements shown in the dashed lines. There was no observed systematic difference in estimated T_2_ for a range of TR, and therefore TR = 4 s is used for SL‐TRUST

Figure 3 demonstrates that despite saturating the spins in the superior sagittal sinus, and therefore delaying the incoming signal, TI = 1200 ms is still an appropriate inversion delay choice for SL‐TRUST. Similarly, Figure 4 shows no systematic differences observed in the measured T_2_ values for TR = 4–8 s for SL‐TRUST and therefore TR = 4 s can be used to minimize the scan time.

During the reproducibility study, only 1 subject failed to complete all of the 4 scan sessions within a 2‐week period. For this subject, only 3 complete scans were acquired and used in subsequent analyses. Additionally, only a single T_1_ measurement was obtained for this subject.

**Figure 5:**
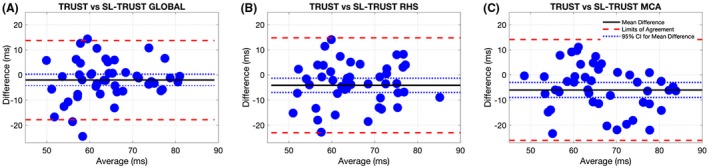
Bland‐Altman correlation plots for T_2_‐measurements using TRUST vs global (A), hemispherical (B), and regional (MCA) SL‐TRUST (C). The solid line (black) is the mean difference between the 2 measurements and the dashed lines indicated the 95% confidence interval (blue) and the agreement limit (red). A slight negative bias of around 5 ms is observed between TRUST and hemispherical (RHS) and regional (MCA) SL‐TRUST

Bland‐Altman plots comparing TRUST and SL‐TRUST T_2_ values in Figure 5 show that there is no significant systematic bias between TRUST and global SL‐TRUST, however, there is a slight systematic bias between TRUST and regional SL‐TRUST T_2_ measurements. The largest deviation in the mean difference is ~5 ms, and the 95% confidence intervals do not contain 0 that indicates an overall significant difference between the 2 measurements.

**Figure 6:**
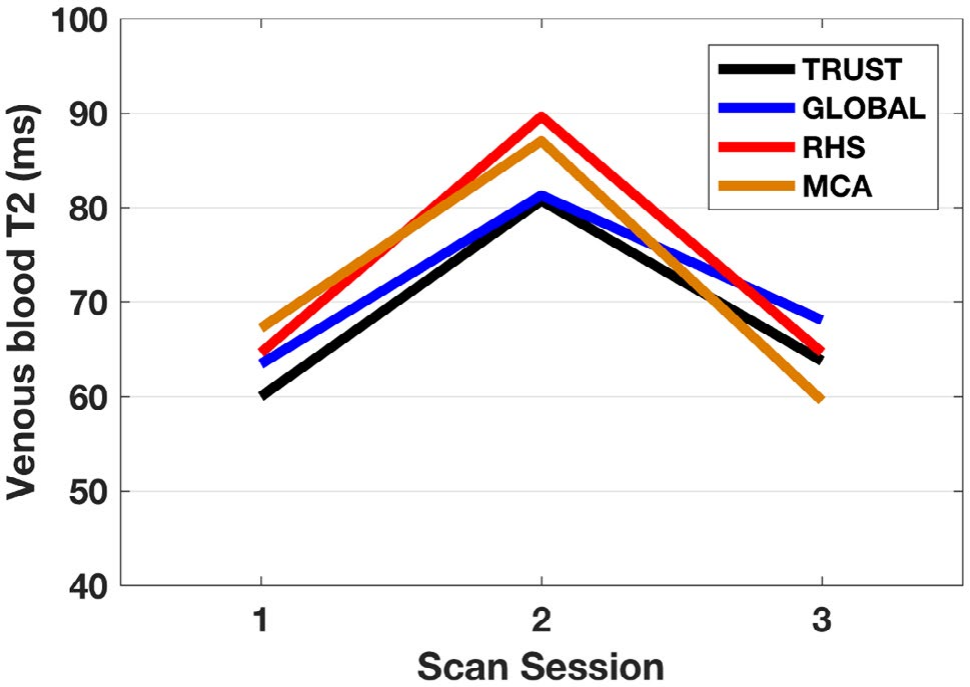
Example of T_2_ variation across multiple days for a single subject (subject 3), with the same effect observed across all scan types indicating that sensitivity to changes in oxygenation is preserved in the regional SL‐TRUST technique

In Figure 6, an example is shown of how the T_2_ was observed to differ in a systematic way between scan sessions within a single participant. This may suggest evidence of SL‐TRUST maintaining the sensitivity of TRUST to natural variation in T_2_ across sessions. In this subject, an increase of 19.05 ms is observed from session 1 to session 2 using TRUST, and increases of 16.36, 22.80, and 18.05 ms for each of the 3 SL‐TRUST measurements. This corresponds to a 10.1% drop in OEF identified using TRUST and an average 9.5% decrease in OEF identified using SL‐TRUST. This is similar in magnitude to the change in oxygenation observed using TRUST following a caffeine challenge,^12^ 7.0 ± 1.8%, which arises because of vasoconstriction effects.

**Figure 7:**
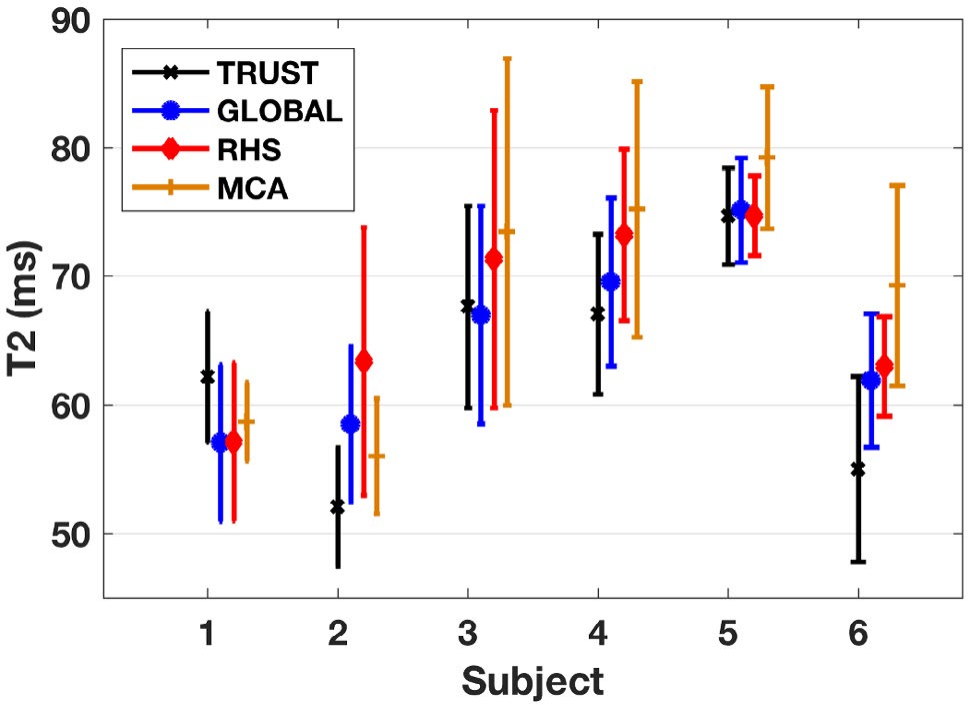
Average and SD of T_2_ values across all scan sessions for each of the 6 subjects for TRUST and global and regional SL‐TRUST

Looking more broadly at the spread of T_2_ values, Figure 7 shows the average and SD of the T_2_ measurement for each scan type and for each subject, taken across all scan sessions. Inter‐subject differences can be observed across all techniques with some participants showing consistently higher (or lower) T_2_ values. The average T_2_ in the sagittal sinus was found to be 60.8 ± 8.9, 62.7 ± 7.9, 64.6 ± 8.4, and 66.3 ± 10.3 ms (mean ± SD) for conventional TRUST, global SL‐TRUST, hemispheric SL‐TRUST, and 3D‐localized SL‐TRUST, respectively. Table 1 provides an insight into the T_2_ and T_1_ values obtained during a single scan session, along with the corresponding estimates of HCT and the resulting OEF. No systematic change in HCT was observed between the 2 gender groups. The T_1_ values are in some cases slightly longer than expected, and as a result, 2 of the HCT values are outside the expected physiological range (<0.3).^36^ In these cases, the OEF values are higher than expected (>50%), that is driven by these lower HCT values. Averaging across all subjects and all scans the average OEF was found to be 45.3 ± 9.0%, 44.2 ± 7.3%, 43.1 ± 7.4%, and 42.5 ± 7.9% for conventional TRUST, global SL‐TRUST, hemispheric SL‐TRUST, and 3D‐localized SL‐TRUST, respectively. Similarly, we calculated the average OEF using uniform HCT values of 0.4 and 0.43 for female and male subjects to give an OEF of 40.6 ± 5.2%, 39.3 ± 4.5%, 38.3 ± 4.6%, and 37.5 ± 5.5% for conventional TRUST, global SL‐TRUST, hemispheric SL‐TRUST, and 3D‐localized SL‐TRUST, respectively. Tables with subject‐specific OEF values using HCT = 0.4 and 0.43 are provided in Supporting Information Table S1.

**Table 1:** T_1_, HCT, and T_2_ for all six subjects, measured in a single scan session, and corresponding OEF

Subject no. (F/M)	T_1_ (ms)	HCT	T_2_ (ms) (±SD)				OEF (%)			
			TRUST	SL‐TRUST			TRUST	SL‐TRUST		
			GLOBAL	GLOBAL	RHS	MCA	GLOBAL	GLOBAL	RHS	MCA
1 (F)	1715.7 ± 74.4	0.447 ± 0.017	64.0 ± 1.6	50.7 ± 1.1	67.4 ± 7.3	53.8 ± 3.9	36.8 ± 1.4	44.9 ± 1.7	35.0 ± 2.3	42.8 ± 2.2
2 (F)	2089.3 ± 95.4	0.298 ± 0.014	51.1 ± 1.1	45.7 ± 9.0	54.8 ± 5.0	56.7 ± 3.7	55.3 ± 2.7	59.2 ± 6.5	53.0 ± 3.5	51.8 ± 3.0
3 (M)	1562.4 ± 85.5	0.529 ± 0.023	61.3 ± 3.8	65.3 ± 3.3	62.2 ± 3.5	57.4 ± 3.4	36.9 ± 1.9	34.6 ± 1.7	36.4 ± 1.9	39.1 ± 2.0
4 (M)	1801.4 ± 68.3	0.407 ± 0.014	65.9 ± 3.6	60.5 ± 0.8	62.0 ± 1.2	61.8 ± 4.2	37.6 ± 1.6	40.6 ± 1.4	39.7 ± 1.4	39.7 ± 1.9
5 (F)	2026.0 ± 67.6	0.319 ± 0.011	70.8 ± 3.6	76.1 ± 2.2	68.6 ± 3.4	76.6 ± 1.9	42.4 ± 1.8	40.2 ± 1.5	43.4 ± 1.8	40.0 ± 1.4
6 (M)	2160.6 ± 113.5	0.275 ± 0.016	58.2 ± 1.0	58.1 ± 3.0	57.0 ± 2.5	63.6 ± 1.1	53.8 ± 3.1	53.9 ± 3.4	54.5 ± 3.3	51.0 ± 2.9

Abbreviations: HCT, hematocrit.

Subject‐specific T_1_ and corresponding HCT values for venous blood along with the T_2_ and OEF values for both TRUST and whole‐brain and regional SL‐TRUST measurements acquired in the same scan session. (±estimated error).

Table 2 shows the intra‐ and inter‐session, and inter‐subject coefficients of variation for both T_2_ and OEF for TRUST and SL‐TRUST. The intra‐ and inter‐session CoV for global OEF measured using SL‐TRUST is found to be 3.00% and 5.98%, which is better than those found for TRUST, 3.25% and 6.76%. A similar accuracy is found for spatially localized SL‐TRUST measurements despite the reduced signal. As predicted the inter‐session CoV is larger than the intra‐session CoV because of subject repositioning and day‐to‐day physiological differences. The variation because of these differences was calculated to be on average 5.50% across all techniques. The inter‐subject CoV on T_2_ for TRUST and SL‐TRUST are all <15% with the exception of SL‐TRUST MCA that is slightly higher at 17.01%. The inter‐subject CoV for OEF, however, is on average around 5% higher. This is because of the additional variance introduced by the subject‐specific HCT measurement obtained from T_1_. This is verified by the calculation of the inter‐subject CoV for OEF using uniform HCT of HCT = 0.4 and 0.43 that gives 12.07%, 12.10%, 12.9%, and 15.55% for conventional TRUST, global SL‐TRUST, hemispheric SL‐TRUST, and 3D‐localized SL‐TRUST, respectively. The additional variance because of inter‐subject physiological differences is calculated to be 18.76%. The intra‐ and inter‐session CoV for T_1_ were 2.54% and 4.71%, respectively.

**Table 2:** Coefficients of Variation for T_2_, OEF, and T_1_ using Equations (7), (8), (9)

CoV (%)	TRUST		GLOBAL		RHS		MCA		T_1_
	T_2_	OEF	T_2_	OEF	T_2_	OEF	T_2_	OEF	
Intra‐session	4.70	3.25	4.24	3.00	5.48	4.38	4.27	3.79	2.54
Inter‐session	8.71	6.76	7.50	5.98	8.37	6.79	8.33	6.84	4.71
Inter‐subject	14.04	20.96	13.80	19.34	13.62	20.03	17.01	19.23	15.8

Abbreviations: CoV, coefficients of variation.

Summary of the intra‐session, inter‐session, and inter‐subject CoV for both T_2_ and OEF across both TRUST and global and regional SL‐TRUST. CoV in the T_1_ measurements is also shown, however, note that the inter‐session T_1_ measurements were all acquired on the same day. As a consequence, the inter‐session variability in T_1_ of 4.71% is artificially low in comparison to that for T_2_, ~7%, where the inter‐session variability is calculated across different days.

## 4 DISCUSSION

In this work, we propose a new method to obtain localized measurements of oxygen extraction fraction non‐invasively. By performing T_2_‐encoding before an inversion labeling pulse, we sensitize the signal of interest to the T_2_ of the blood while it is still in the venules and/or veins, before reading out in the larger sagittal sinus draining vein. This circumvents the problem of measuring the T_2_ of the blood in the sagittal sinus itself, which would only report on the global (non‐spatially averaged) OEF.

An inversion delay TI of 1200 ms was used for both TRUST and SL‐TRUST in this healthy cohort, however, we expect regional SL‐TRUST to be more sensitive to differences in cerebral blood flow (CBF) that are commonly found in clinical cohorts (e.g., stroke patients). Therefore, in these populations, some further calibrations would likely be required to determine the inversion time accordingly and maximize signal from specific brain regions. The risk of longer inversion delays is that the measured signal may become sensitive to the T_2_ of capillary or tissue water, rather than blood that is already in the draining microveins of the tissue region, something that would need to be investigated further.

A short study into the optimum TR time of the SL‐TRUST method indicates that we do not see the same systematic underestimation of T_2_ at shorter TR values that has been observed for TRUST.^29^ This is likely because of the variety of spatial saturation pulses and background suppression pulses, which, when combined, serve a similar purpose as the post‐saturation pulse used in later versions of TRUST, to reset the magnetisation.^29^

Effective saturation of blood signal from unwanted tissue is critical for the successful isolation of blood from specific brain regions. This saturation must also be rapid compared to the timescale over which blood spins flow out of the region of tissue that is being saturated, therefore the saturation scheme must also be reasonably short in duration. Initially, the WET saturation scheme does not appear to be ideal in this respect because of the use of 4 consecutive RF pulses. However, the duration of each WET module is still relatively short (40 ms), and any outflow effects during this time are outweighed by the superior saturation profile achieved compared to standard saturation pulses (data not shown).^20^, ^21^ Additionally, the summation of 2 pulses with a short time shift allowed twice the number of saturation regions to be played out with a modest 25% increase in the overall saturation duration.

Regional OEF values for both a single hemisphere and a 70 × 70 × 80 mm^3^ region in the brain have been obtained with similar precision and reproducibility compared to whole‐brain conventional TRUST measurements, suggesting that the dominant source of noise is of physiological origin, and therefore scales correspondingly with the signal from smaller tissue regions. Although the global TRUST and SL‐TRUST T2 values obtained are not significantly different, we do observe a small but significant difference between TRUST and regional SL‐TRUST T2 values. This systematic bias of 5 ms between TRUST and SL‐TRUST translates into an ~3% difference in OEF between the 2 methods. In comparison, the QUIXOTIC method, which is perhaps the closest technique to SL‐TRUST in terms of spatial and temporal resolution, has reported a systematic bias of up to 10% difference in OEF in comparison to TRUST.^10^ In terms of the SL‐TRUST technique, this small change might be expected given that the blood has originated from different parts of the brain that may be more sensitive to changes in factors such as activation or fatigue. There is also some evidence that the sensitivity to changes in physiology has been preserved in SL‐TRUST (Figure 6).

Overall, good agreement was found between the reproducibility of OEF measurements from TRUST and SL‐TRUST found in this study, with those from a previous TRUST reproducibility study by Liu et al.,^35^ where the intra‐session, inter‐session, and inter‐subject CoV was found to be 3.19%, 8.16%, and 15.61% respectively.

The inclusion of an estimate of individual HCT values, via a non‐invasive blood T_1_ measurement, would be particularly valuable where red blood cell or hemoglobin content is expected to be different (e.g., in disease populations or in neonates).^37^, ^38^ Over a physiologically accepted range of HCT = 0.38–0.46, the calibration curve given in Equation 1 results in a variation of 4% in OEF, for a blood T_2_ of 68 ms and T_CPMG_ = 10 ms (see Supporting Information Figure S1). Considering fluctuations in OEF have been observed on the scale of 10%, a potential variation of 4% because of HCT indicates that it is an important parameter to include. Although there is some evidence to suggest that in vivo measurements of blood T_1_ are higher than in vitro measurements,^28^, ^39^, ^40^ the large inter‐subject variation observed in our measurements result in HCT values that are outside the physiological range and therefore result in a higher‐than‐anticipated OEF in those subjects. This suggests that further improvements need to be made to the T_1_ measurement, and there exists several alternative methods to measure T_1_, including a Look‐Locker approach for a faster estimation of T_1_, or performing the measurement in the internal jugular vein.^40^, ^41^ Further assessment could also include measuring T_1_ over multiple days to investigate and account for day‐to‐day physiological differences in HCT.

Participant 6 showed the largest difference in the average T_2_ between scan types and hence a larger distribution in OEF. An explanation of this is likely to be motion in the subject, because the largest degree of motion was observed in this participant and the ROI around the superior sagittal sinus had to be re‐defined appropriately between measurements during post‐processing. One possible solution to the difficulties of post‐processing motion correction of these images in 2D is the addition of prospective motion navigators. The sequence lends itself well to the inclusion of motion navigators because of the ample dead time, and indeed, this was investigated by Stout et al^42^ for the conventional TRUST sequence. In that study, a volume navigator was included during the inversion delay and used as a retrospective motion correction tool. They found that under the presence of motion, the T_2_ values were ~7–10 ms longer, a bias that is almost half of the physiological change most studies try to detect.

Another limitation or potential source of variability in the measurements is the assumption that the initial magnetization is at equilibrium at the start of each measurement. Imperfect flip angles in the re‐focusing RF pulses in the T_2_‐encoding scheme can produce stimulated echoes that contribute to the net signal decay, resulting in a systematic overestimation of T_2_.^24^

## 5 CONCLUSION

The SL‐TRUST sequence seeks to address previous limitations of the TRUST method by enabling regionally specific measurements of OEF. WET saturation schemes, optimized for spatial saturation and to allow multiple saturation bands to be performed simultaneously, achieve effective and efficient saturation of venous blood spins in brain tissue outside the ROI. Background suppression methods in the readout slice help mitigate partial volume effects from surrounding static tissue and also help to counteract the effect of moving the T_2_‐encoding earlier in the sequence.

Sensitivity to changes in OEF appear to have been maintained relative to conventional TRUST, however, further studies using gas challenges or patient population groups will help confirm this. Our results suggest that an OEF comparison of 2 contralateral regions can be achieved in under 5 min on a standard 3T system with a CoV of under 5%, comparable to whole brain TRUST and the gold standard of PET O15.^35^ Therefore, SL‐TRUST shows potential to become a reliable, robust, and rapid method of quantifying regional OEF differences. Although there exist MR methods that can achieve higher spatial resolution than SL‐TRUST, these also suffer from unexplained systematic biases in the measurement and can be highly dependent on sequence parameters. Even localizing to individual hemisphere OEF, such as has been achieved here using SL‐TRUST, has been identified as a powerful tool (e.g., as a predictor of future stroke risk in patients with complete carotid artery occlusion).^43^

Overall, SL‐TRUST allows for better understanding of brain physiology in both healthy and clinical populations in a non‐invasive and time‐efficient manner and can be easily performed on clinically available MRI scanners.

## Supporting information


**FIGURE S1** Hematocrit dependence on the calibration between T_2_ blood measurement and calculated venous blood oxygenation level using calibration given in Equation 1
**TABLE S1** Example T_2_ measurements and the resulting OEF values calculated using uniform hematocrit values of 0.4 for female subjects and 0.43 for male subjects
