# Supplementary material for: Volume‐localized measurement of oxygen extraction fraction in the brain using MRI
Source: Magn Reson Med. 2019 May 27;82(4):1412–23. doi: 10.1002/mrm.27823 (PMC6772021; doi:10.1002/mrm.27823)
Supplement: Supplementary file 1 — FIGURE S1 Hematocrit dependence on the calibration between T2 blood measurement and calculated venous blood oxygenation level using calibration given in Equation 1 TABLE S1 Example T2 measurements and the resulting OEF values calculated using uniform hematocrit values of 0.4 for female subjects and 0.43 for male subjects [file MRM-82-1412-s001.docx]

*
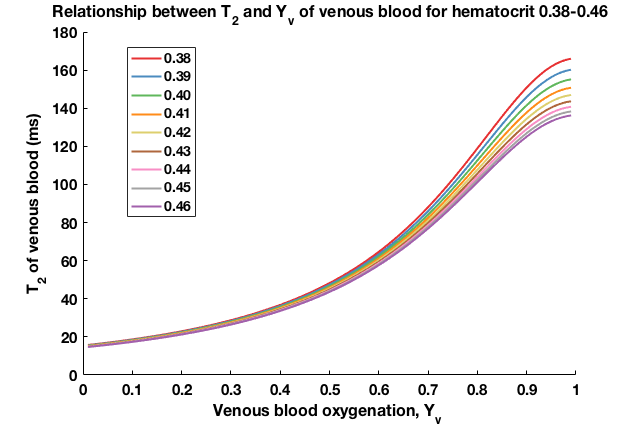
*

Supporting Information Figure S1: Hematocrit dependence on the calibration between T2 blood measurement and calculated venous blood oxygenation level using calibration given in Equation 1.

| **Subject** | **hct** | **T2 (ms) (**$\boldsymbol{\pm}$**std)** | | | | **OEF (%)** | | | |
| --- | --- | --- | --- | --- | --- | --- | --- | --- | --- |
|  |  | **TRUST** | **SL-TRUST** | | | **TRUST** | **SL-TRUST** | | |
| **# (F/M)** |  | GLOBAL | GLOBAL | RHS | MCA | GLOBAL | GLOBAL | RHS | MCA |
| **1 (F)** | 0.4 | 64.0 $\pm$1.6 | 50.7 $\pm$ 1.1 | 67.4 $\pm$ 7.3 | 53.8 $\pm$ 3.9 | 39.0 $\pm$ 1.0 | 47.0 $\pm$ 1.0 | 37.3 $\pm$ 2.7 | 45.0 $\pm$ 4.1 |
| **2 (F)** | 0.4 | 51.1 $\pm$ 1.1 | 45.7 $\pm$ 9.0 | 54.8 $\pm$ 5.0 | 56.7 $\pm$ 3.7 | 46.7 $\pm$ 1.1 | 50.7 $\pm$ 10.6 | 44.3 $\pm$ 3.2 | 43.2 $\pm$ 4.0 |
| **3 (M)** | 0.43 | 61.3 $\pm$ 3.8 | 65.3 $\pm$ 3.3 | 62.2 $\pm$ 3.5 | 57.4 $\pm$ 3.4 | 38.9 $\pm$ 2.4 | 36.8 $\pm$ 1.9 | 38.5 $\pm$ 2.3 | 41.2 $\pm$ 2.5 |
| **4 (M)** | 0.43 | 65.89 $\pm$ 3.6 | 60.5 $\pm$ 0.8 | 62.0 $\pm$ 1.2 | 61.8 $\pm$ 4.2 | 36.4 $\pm$ 2.1 | 39.4 $\pm$ 0.6 | 38.6 $\pm$0.8 | 38.6 $\pm$ 2.6 |
| **5 (F)** | 0.4 | 70.8 $\pm$ 3.6 | 76.1 $\pm$ 2.2 | 68.6 $\pm$ 3.4 | 76.6 $\pm$ 1.9 | 35.7 $\pm$ 2.0 | 33.3 $\pm$ 1.0 | 36.7 $\pm$ 2.0 | 33.1 $\pm$ 1.0 |
| **6 (M)** | 0.43 | 58.2 $\pm$ 1.0 | 58.1 $\pm$ 3.0 | 57.0 $\pm$ 2.5 | 63.6 $\pm$ 1.1 | 40.7 $\pm$ 0.7 | 40.8 $\pm$ 2.2 | 41.4 $\pm$ 1.8 | 37.7 $\pm$ 0.7 |

Supporting Information Table S1: Example T2 measurements and the resulting OEF values calculated using uniform hematocrit values of 0.4 for female subjects and 0.43 for male subjects.
